# Age and morphology of posterior communicating artery aneurysms

**DOI:** 10.1038/s41598-020-68276-9

**Published:** 2020-07-14

**Authors:** Jian Zhang, Anil Can, Pui Man Rosalind Lai, Srinivasan Mukundan, Victor M. Castro, Dmitriy Dligach, Sean Finan, Sheng Yu, Vivian S. Gainer, Nancy A. Shadick, Guergana Savova, Shawn N. Murphy, Tianxi Cai, Scott T. Weiss, Rose Du

**Affiliations:** 1 Department of Neurosurgery, Brigham and Women’s Hospital, Harvard Medical School, 75 Francis Street, Boston, MA 02115 USA; 2 Department of Neurosurgery and Brain and Nerve Research Laboratory, The First Affiliated Hospital of Soochow University, Suzhou, Jiangsu Province China; 3 Department of Radiology, Brigham and Women’s Hospital, Boston, MA USA; 4 Research Information Systems and Computing, Partners Healthcare, Boston, MA USA; 5 Boston Children’s Hospital Informatics Program, Boston, MA USA; 6 Department of Computer Science, Loyola University, Chicago, IL USA; 7 Department of Medicine, Brigham and Women’s Hospital, Boston, MA USA; 8 Center for Statistical Science, Tsinghua University, Beijing, China; 9 Division of Rheumatology, Immunology and Allergy, Brigham and Women’s Hospital, Boston, MA USA; 10 Department of Neurology, Massachusetts General Hospital, Boston, MA USA; 11 Biostatistics, Harvard School of Public Health, Boston, MA USA; 12 Channing Division of Network Medicine, Brigham and Women’s Hospital, Boston, MA USA

**Keywords:** Cerebrovascular disorders, Risk factors

## Abstract

Risk of intracranial aneurysm rupture could be affected by geometric features of intracranial aneurysms and the surrounding vasculature in a location specific manner. Our goal is to investigate the morphological characteristics associated with ruptured posterior communicating artery (PCoA) aneurysms, as well as patient factors associated with the morphological parameters. Three-dimensional morphological parameters in 409 patients with 432 PCoA aneurysms diagnosed at the Brigham and Women’s Hospital and Massachusetts General Hospital between 1990 and 2016 who had available CT angiography (CTA) or digital subtraction angiography (DSA) were evaluated. Morphological parameters examined included aneurysm wall irregularity, presence of a daughter dome, presence of hypoplastic or aplastic A1 arteries and hypoplastic or fetal PCoA, perpendicular height, width, neck diameter, aspect and size ratio, height/width ratio, and diameters and angles of surrounding parent and daughter vessels. Univariable and multivariable statistical analyses were performed to determine the association of morphological parameters with rupture of PCoA aneurysms. Additional analyses were performed to determine the association of patient factors with the morphological parameters. Irregular, multilobed PCoA aneurysms with larger height/width ratios and larger flow angles were associated with ruptured PCoA aneurysms, whereas perpendicular height was inversely associated with rupture in a multivariable model. Older age was associated with lower aspect ratio, with a trend towards lower height/width ratio and smaller flow angle, features that are associated with a lower rupture risk. Morphological parameters are easy to assess and could help in risk stratification in patients with unruptured PCoA aneurysms. PCoA aneurysms diagnosed at older age have morphological features associated with lower risk.

## Introduction

While general features such as size is known to affect the rupture risk of an aneurysm, the effects of more specific morphological features, as well as acquired risk factors such as smoking and hypertension, in a site specific manner remains to be elucidated^1,2^. Hemodynamic stress has been shown to be affected by morphological parameters of the aneurysm and the surrounding vascular anatomy^3–15^. Therefore, investigating the effects of morphological parameters of the aneurysm and surrounding vascular tree that affect these hemodynamic factors in a location specific manner would be important in understanding the rupture risk of an aneurysm. Here, we present a large sample of 432 posterior communicating artery (PCoA) aneurysms that were examined using a diverse series of morphological and clinical variables to assess the features that are associated with rupture. Our study is unique in the large number of aneurysms, the inclusion of parameters that involve the surrounding vascular anatomy which are not intrinsic to aneurysm morphology, and the evaluation of clinical factors that may be associated with particular morphologies.

## Methods

### Patient selection

**Figure 1:**
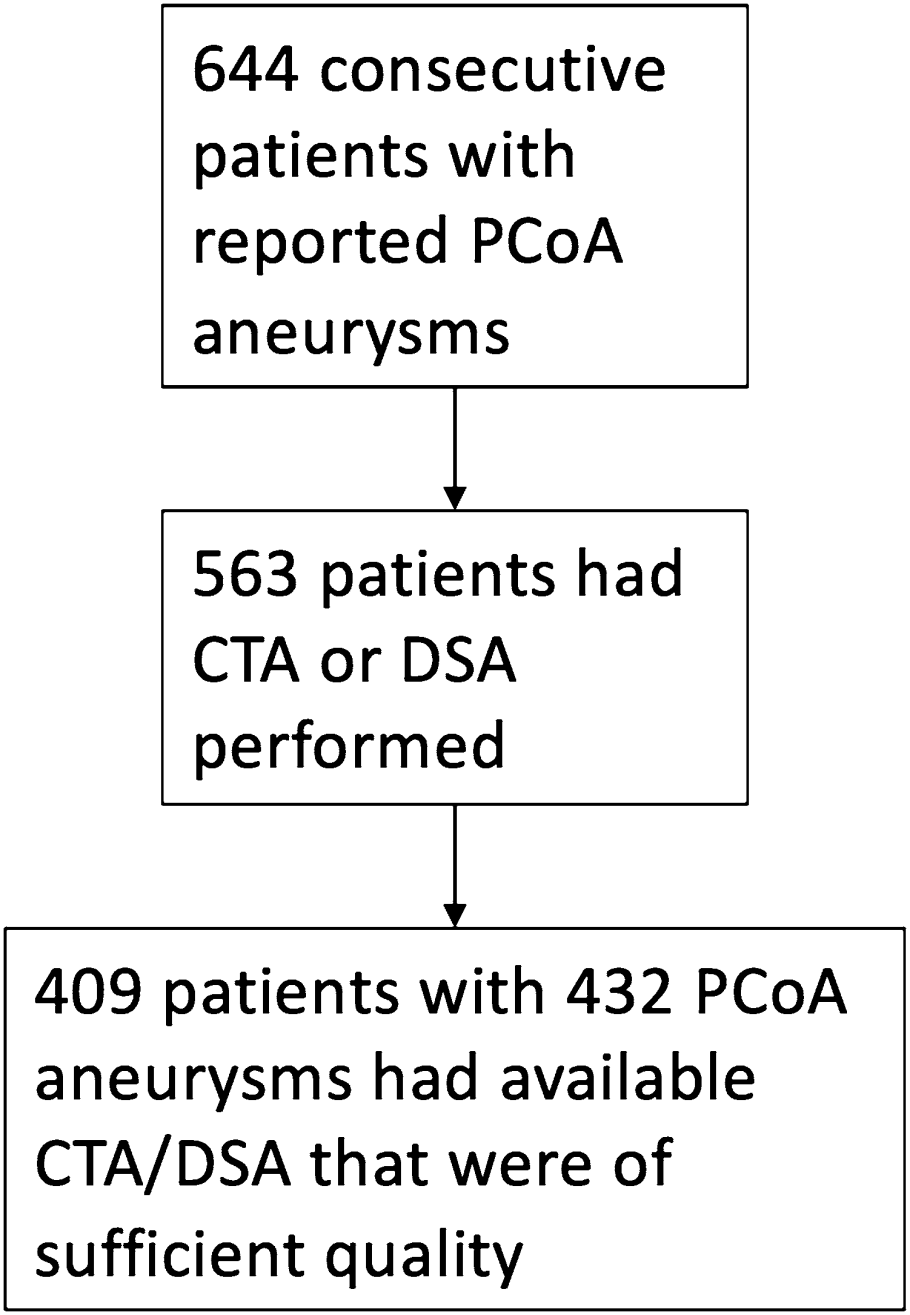
Flow chart for patient selection.

Using natural language processing (NLP) in conjunction with manual medical record review from the Partners Healthcare Research Patients Data Registry (RPDR), patients diagnosed with an intracranial aneurysm at the Brigham and Women’s Hospital (BWH) and Massachusetts General Hospital (MGH), from 1990 to 2016 were identified. The RPDR includes 4.2 million patients who have received care from BWH and MGH. Using a machine learning algorithm based on both codified and NLP data to identify an initial set of patients with potential aneurysms from the RPDR, 5,589 patients were eventually identified^16^, of which 727 patients were also seen on clinical presentation from 2007 to 2013 with prospectively collected data. An additional 474 patients with prospectively collected data who were seen on clinical presentation from 2013–2016, were also included, resulting in a total of 6,063 patients. 4,701 patients with definite saccular aneurysms were identified by manually reviewing (AC and RD) the medical records of all 6,063 patients^17^. 644 consecutive patients had reported posterior communicating artery aneurysms. Of those, 563 had CT angiography (CTA) or digital subtraction angiography (DSA) performed. 409 patients with 432 posterior communicating artery (PCoA) aneurysms had available imaging of sufficient quality which were obtained using mi2b2 open-source software to comply with research privacy requirements (Fig. 1)^18^. Only saccular aneurysms were included. Aneurysms associated with arteriovenous malformations and fusiform aneurysms were excluded from this study. Demographic and clinical information, including tobacco and alcohol use, history of hypertension, and family history of intracranial aneurysms and subarachnoid hemorrhage, was retrieved from medical records. This study was approved by the Partners Institutional Review Board which waived the requirement for informed consent. All procedures performed were in accordance with the ethical standards of the institutional review board and with the 1964 Helsinki declaration and its later amendments or comparable ethical standards.

### Reconstruction of 3D models

Using preoperative CTA via the Vitrea Advanced Visualization software (version 6.9.68.1, Vital Images, Minnetonka, MN), three-dimensional (3D) models of aneurysms and their surrounding vasculature were generated. The software creates a spatial reconstruction of the vasculature from axial CTA images in the DICOM (Digital Images and Communication in Medicine) format. DSA studies with 3D reconstructions were evaluated directly. We manually measured lengths and angles. In order to ensure accurate measurements, windowing for the 3D reconstructions were validated against the multiplanar reconstructions.

### Definition of morphological parameters

**Figure 2:**
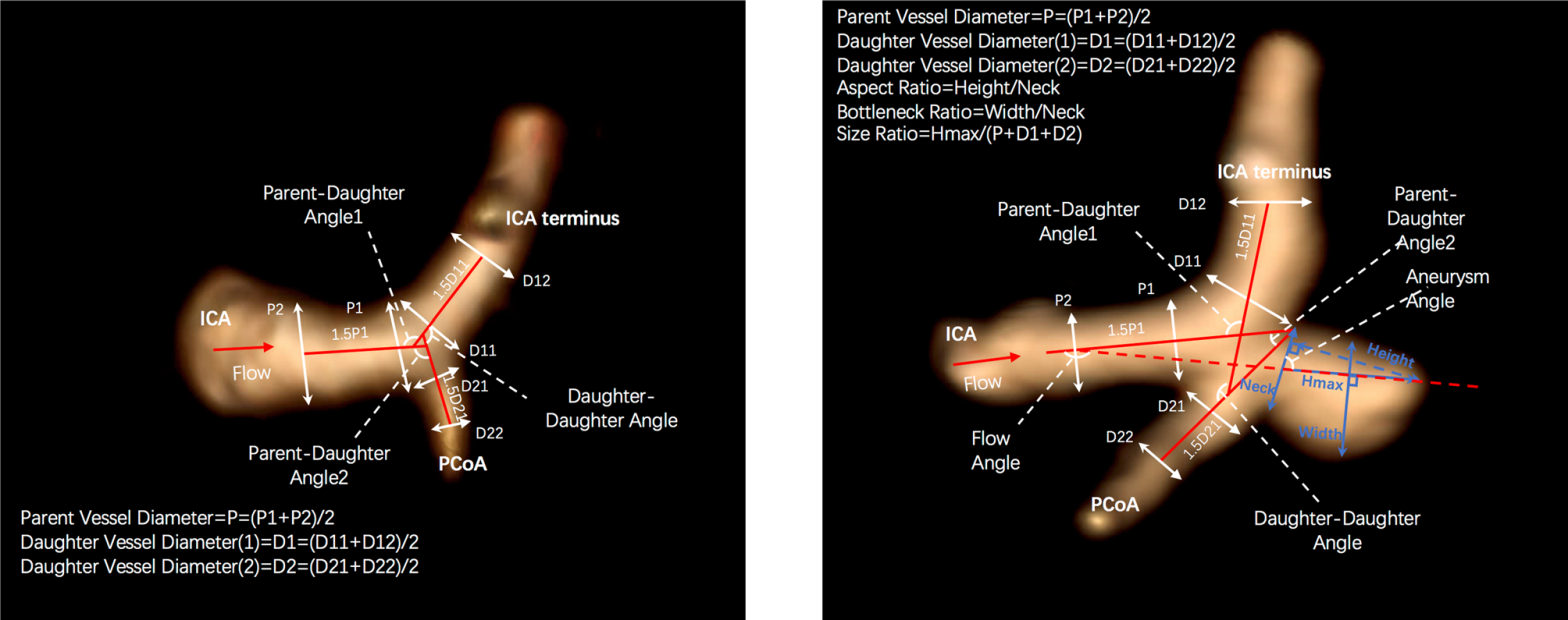
Illustrations of morphological parameters.

Both aneurysm related variables and measurements of the surrounding vasculature were used in our study, and are described briefly below (Fig. 2). PCoA aneurysms were categorized as smooth or irregular (non-smooth wall), and with or without daughter domes. If hypoplastic A1s, aplastic A1s, hypoplastic/aplastic posterior communicating arteries (PCoAs), and/or fetal PCoAs were present, the side of the anatomical variation was noted (e.g. ipsilateral or contralateral to the PCoA aneurysm). An A1 was considered hypoplastic if its diameter was less than half of the contralateral A1. A PCoA was considered hypoplastic/aplastic if it was not visible on CTA. Maximum aneurysm height was defined as the length between the center of the aneurysm neck and the greatest distance to the dome, whereas maximum perpendicular height was the largest perpendicular distance from the neck of the aneurysm to the dome of the aneurysm. In addition, we measured the neck diameter, the width of the aneurysm (maximal diameter perpendicular to maximum height line), and the aspect ratio (AR) which was calculated as the ratio of the maximum perpendicular height of the aneurysm to the average neck diameter of the aneurysm. Height/width ratio was defined as the ratio of maximum perpendicular height to width. Size ratio was calculated by dividing the maximum height by the mean vessel diameter of all branches (parent and daughter arteries) associated with the aneurysm. Vessel diameters were measured by averaging the diameter of the cross-section of a vessel (D) just proximal to the neck of the aneurysm and the diameter of the cross-section at 1.5 times D from the neck of the aneurysm. Average diameters of the parent artery, larger daughter branch and the smaller daughter branch (PCoA) were calculated in this manner. The diameter size ratio was defined as the parent artery diameter divided by the sum of the diameters of both daughter branches, and the daughter diameter ratio was defined as the larger daughter artery diameter divided by the smaller daughter artery diameter. Daughter-daughter angle was defined as the angle formed between the daughter vessels, parent-daughter angle was the angle between the parent vessel and the daughter vessel, and the flow angle was the angle between the maximum height of the aneurysm and the parent vessel.

### Statistical analysis

We evaluated differences in baseline characteristics between the ruptured and unruptured groups using the *t-*test for continuous variables and the Pearson's chi-square test for categorical variables. Univariable and multivariable logistic regression models were used to test for effects of different morphological parameters on rupture status, with a backward elimination procedure to identify significant confounders. The effects of patient characteristics on aneurysm morphology was also examined using univariable and multivariable regression models. We used cut-off values of 0.1 in order to select the initial set of variables to be included in the initial multivariable model for backward elimination. Adjusted odds ratios (OR) with 95% confidence intervals (CIs) were calculated and *P* < 0.05 was considered significant. All statistical analyses were performed using the Stata statistical software package (version 14, StataCorp. College Station, TX).

## Results

409 patients with 432 PCoA aneurysms were included in this study. Table 1 shows the demographic and clinical information of the study population. The mean patient age was 58.8 ± 14.5 years, and 83.4% of patients were female. Patients with ruptured aneurysms were younger (56.7 ± 15 vs. 61.8 ± 13 years), more frequently tobacco users (46% vs 34%), and were less likely to have a family history of intracranial aneurysms or subarachnoid hemorrhage (5% vs 11%). In addition, patients with ruptured aneurysms were more frequently alcohol users and were more likely to have hypertension, although the differences were not statistically significant.

**Table 1:** Demographic information and clinical risk factors of patients with ruptured and unruptured aneurysms (N = 409).

Variables	All patients (N = 409)	Patients with ruptured aneurysms (N = 237)	Patients with unruptured aneurysms (N = 172)	*P*-value
Age (SD)	58.8 (14.5)	56.7 (15.2)	61.8 (12.8)	< 0.01
Female (%)	326 (83.4)	187 (81.7)	139 (85.8)	0.28
Alcohol use (current) (%)	172 (48.0)	99 (48.1)	73 (48.0)	0.99
Tobacco use (current) (%)	157 (41.2)	103 (46.4)	54 (34.0)	0.02
Hypertension (%)	200 (51.2)	120 (52.4)	80 (49.4)	0.56
Family history of SAH (%)	30 (7.7)	12 (5.2)	18 (11.1)	0.03
Family history of aneurysms	51 (13.0)	22 (9.6)	29 (17.9)	0.02

We then examined the predefined morphological parameters of the aneurysms in the ruptured and unruptured groups (Table 2). Ruptured aneurysms had a higher rate of irregularity (69% vs 22%), daughter dome (80% vs 23%), greater perpendicular height (6.0 vs 5.0 mm), maximum height (6.7 vs 5.5 mm), aspect ratio (1.9 vs 1.4), height/width ratio (1.3 vs 1.1), size ratio (0.95 vs 0.74), and flow angle (119 vs 108 degrees), non-hypoplastic PCoA (63% vs 51%), and smaller distal ICA diameter (3.2 vs 3.4 mm), proximal ICA diameter (3.0 vs 3.2 mm), and diameter size ratio (0.71 vs 0.75). There was no difference in the side of aneurysm, fetal PCoA variant, PCoA diameter, hypoplastic or aplastic A1, neck diameter, width of aneurysm, daughter-daughter angle, and parent-daughter angle ratio between the ruptured and unruptured groups.

**Table 2:** Aneurysm characteristics stratified by rupture status of PCoA aneurysms (N = 432).

Variables	All (N = 432)	Missing	Ruptured (N = 239)	Unruptured (N = 193)	*P*-value
Side					
Left (%)	165 (38.2)	0	84 (35.1)	81 (42.0)	0.14
Right (%)	267 (61.8)	0	155 (64.9)	112 (58.0)	0.14
Irregular (%)	207 (47.9)	0	165 (69.0)	42 (21.8)	< 0.01
Daughter dome (%)	236 (54.6)	0	192 (80.3)	44 (22.8)	< 0.01
Hypoplastic/aplastic PCoA (%)					
No	250 (57.9)	0	151 (63.2)	99 (51.3)	0.01
Ipsilateral	57 (13.2)	0	28 (11.7)	29 (15.0)	0.31
Contralateral	77 (17.8)	0	37 (15.5)	40 (20.7)	0.16
Both	48 (11.1)	0	23 (9.6)	25 (13.0)	0.26
Fetal PCoA (%)					
No	252 (58.3)	0	141 (59.0)	111 (57.5)	0.75
Ipsilateral	102 (23.6)	0	53 (22.2)	49 (25.4)	0.44
Contralateral	35 (8.1)	0	21 (8.8)	14 (7.3)	0.57
Both	43 (10.0)	0	24 (10.0)	19 (9.8)	0.94
Hypoplastic A1 (%)					
No	402 (93.1)	0	227 (95.0)	175 (90.7)	0.08
Ipsilateral	9 (2.1)	0	3 (1.3)	6 (3.1)	0.20
Contralateral	21 (4.9)	0	9 (3.8)	12 (6.2)	0.25
Aplastic A1 (%)					
No	425 (98.4)	0	235 (98.3)	190 (98.4)	0.94
Ipsilateral	3 (0.7)	0	1 (0.4)	2 (1.0)	0.45
Contralateral	4 (0.9)	0	3 (1.3)	1 (0.5)	0.39
Maximum height in mm (SD)	6.15 (3.07)	0	6.70 (2.88)	5.46 (3.17)	< 0.01
Perpendicular height in mm (SD)	5.57 (2.80)	0	6.04 (2.80)	4.98 (2.69)	< 0.01
Neck diameter in mm (SD)	3.45 (1.28)	0	3.38 (1.31)	3.52 (1.23)	0..27
Width in mm (SD)	4.94 (2.61)	0	5.05 (2.67)	4.80 (2.53)	0.32
Aspect ratio (SD)	1.67 (0.73)	0	1.86 (0.73)	1.44 (0.66)	< 0.01
Height/width ratio	1.21 (0.59)	0	1.31 (0.71)	1.07 (0.34)	< 0.01
Average diameter of larger daughter branch (distal ICA) in mm (SD)	3.25 (0.61)	0	3.16 (0.61)	3.35 (0.59)	< 0.01
Average diameter of smaller daughter branch (PCoA) in mm (SD)	1.51 (0.51)	111	1.51 (0.52)	1.51 (0.50)	0.93
Daughter diameter ratio (larger/smaller) (SD)	2.29 (0.89)	111	2.29 (0.89)	2.48 (1.13)	0.09
Parent artery (ICA) diameter in mm (SD)	3.10 (0.60)	0	3.00 (0.58)	3.21 (0.61)	< 0.01
Diameter size ratio (Parent/(D1 + D2))	0.73 (0.15)	0	0.71 (0.15)	0.75 (0.14)	0.01
Size ratio (SD)	0.86 (0.46)	0	0.95 (0.47)	0.74 (0.42)	< 0.01
Daughter–daughter angle in degrees (SD)	144.6 (16.9)	111	144.2 (18.0)	145.2 (15.3)	0.59
Parent–daughter angle ratio (SD)	1.73 (0.71)	111	1.68 (0.65)	1.81 (0.79)	0.11
Flow angle in degrees (SD)	114.3 (20.8)	0	119.2 (20.0)	108.2 (20.3)	< 0.01

Table 3 shows the results of the univariable and multivariable analyses for rupture status of the PCoA aneurysms. In the univariable analysis, irregularity (OR 8.0, 95% CI 5.2–12.4), presence of a daughter dome (OR 13.8, 95% CI 8.7–22.0), larger flow angle (OR 1.03, 95% CI 1.02–1.04), larger maximum height (OR 1.16, 95% CI 1.08–1.25), larger perpendicular height (OR 1.16, 95% CI 1.08–1.26), larger aspect (height/neck) ratio (OR 2.51, 95% CI 1.83–3.45), larger height/width ratio (OR 4.0, 95% CI 2.33–6.88), and larger size ratio (OR 3.37, 95% CI 2.04–5.56) were significantly associated with aneurysm rupture. In addition, diameter of the larger daughter branch (distal ICA) (OR 0.60, 95% CI 0.43–0.82), parent artery (proximal ICA) diameter (OR 0.56, 95% CI 0.40–0.77) and diameter size ratio (OR 0.18, 95% CI 0.05–0.71) were significantly and inversely associated with ruptured status. In multivariable analysis, irregular aneurysm shape (OR 3.25, 95% CI 1.82–5.80), presence of a daughter dome (OR 10.12, 95% CI 5.49–18.65), larger height/width ratio (OR 5.63, 95% CI 2.40–13.22) and larger flow angle (OR 1.03, 95% CI 1.02–1.004) were significantly associated with rupture status. In contrast, perpendicular height (OR 0.85, 95% CI 0.73–0.98) was significantly inversely associated with ruptured PCoA aneurysms. Note that this inverse relationship for perpendicular height is due to the presence of the association of rupture with larger height/width ratio.

**Table 3:** Univariable and multivariable logistic regression for rupture status (N = 432).

Variables	Univariable		Multivariable	
	OR (95% CI)	*P*-val	OR (95% CI)	*P*-val
Side				
Right (vs. left)	1.33 (0.90–1.97)	0.15	–	–
Irregular	8.02 (5.17–12.43)	< 0.01	3.25 (1.82–5.80)	< 0.01
Daughter dome	13.83 (8.70–22.00)	< 0.01	10.12 (5.49–18.65)	< 0.01
Hypoplastic/aplastic PCoA (%)				
Ipsilateral (vs. no)	0.63 (0.36–1.13)	0.12	–	–
Contralateral (vs. no)	0.61 (0.36–1.01)	0.06	–	–
Both (vs. no)	0.60 (0.32–1.12)	0.11	–	–
Fetal PCoA (%)				
Ipsilateral (vs. no)	0.85 (0.54–1.35)	0.50	–	–
Contralateral (vs. no)	1.18 (0.57–2.43)	0.65	–	–
Both (vs. no)	0.99 (0.52–1.91)	0.99	–	–
Hypoplastic A1 (%)				
Ipsilateral (vs. no)	0.39 (0.10–1.56)	0.18	–	–
Contralateral (vs. no)	0.58 (0.24–1.40)	0.23	–	–
Aplastic A1 (%)				
Ipsilateral (vs. no)	0.40 (0.04–4.49)	0.46	–	–
Contralateral (vs. no)	2.43 (0.25–23.51)	0.44	–	–
Maximum height in mm (SD)	1.16 (1.08–1.25)	< 0.01	–	–
Perpendicular height in mm (SD)	1.16 (1.08–1.26)	< 0.01	0.85 (0.73–0.98)	0.03
Diameter neck in mm (SD)	0.92 (0.79–1.07)	0.27	–	–
Width aneurysm in mm (SD)	1.04 (0.96–1.12)	0.32	–	–
Aspect ratio (SD)	2.51 (1.83–3.45)	< 0.01	1.36 (0.72–2.57)	0.35
Height/width ratio	4.00 (2.33–6.88)	< 0.01	5.63 (2.40–13.22)	< 0.01
Average diameter of larger daughter branch (distal ICA) in mm (SD)	0.60 (0.43–0.82)	< 0.01	–	–
Average diameter of smaller daughter branch (PCoA) in mm (SD)	1.02 (0.66–1.58)	0.93	–	–
Daughter diameter ratio (larger/smaller) (SD)	0.83 (0.66–1.03)	0.10	–	–
Parent artery (ICA) diameter in mm (SD)	0.56 (0.40–0.77)	< 0.01	–	–
Diameter size ratio (Parent/(D1 + D2))	0.18 (0.05–0.71)	0.01	–	–
Size ratio (SD)	3.37 (2.04–5.56)	< 0.01	–	–
Daughter–daughter angle in degrees (SD)	1.00 (0.98–1.01)	0.59	–	–
Parent–daughter angle ratio (SD)	0.77 (0.56–1.07)	0.12	–	–
Flow angle in degrees (SD)	1.03 (1.02–1.04)	< 0.01	1.03 (1.02–1.04)	< 0.01

Additional analyses were performed to determine patient factors that were associated with each morphological parameter that was significantly associated with rupture (Table 4, Supplementary Table 1). In the univariable analyses, smoking history was associated with a larger aspect ratio (β = 0.16, 95% CI 0.02–0.30) and larger height/width ratio (β = 0.16, 95% CI 0.04–0.27). Age was inversely associated with aspect ratio (β = − 0.01, 95% CI − 0.02 to − 0.01), height/width ratio (β = − 0.01, 95% CI − 0.01 to − 0.002), flow angle (β = − 0.19, 95% CI − 0.32 to − 0.05), presence of irregularity (OR 0.98, 95% CI 0.97–0.99), and presence of daughter dome (OR 0.99, 95% CI 0.97–1.0). Family history was inversely associated with perpendicular height (β = − 0.82, 95% CI − 1.6 to − 0.03). In the multivariable analyses, age remained inversely associated with aspect ratio (β = − 0.01, 95% CI − 0.01 to − 0.05) with a trend towards an inverse association with height/width ratio and flow angle. There was also a trend towards an association of smoking with height/width ratio and an inverse association of family history with perpendicular height.

**Table 4:** Univariate and multivariate regressions for morphological parameters.

Variables	Univariable		Multivariable	
	Coef (95% CI)	*P*-value	Coef (95% CI)	*P*-value
*Aspect ratio*				
Age at diagnosis	− 0.01 (− 0.02 to − 0.01)	< 0.01	− 0.01 (− 0.01 to 0.05)	< 0.01
Alcohol use	− 0.04 (− 0.18 to 0.10)	0.58	–	–
Tobacco use	0.16 (0.02 to 0.30)	0.03	0.06 (− 0.07 to 0.20)	0.36
Female	0.003 (− 0.18 to 0.19)	0.97	–	–
Hypertension	− 0.09 (− 0.23 to 0.05)	0.21	–	–
Family history aneurysm	− 0.04 (− 0.25 to 0.16)	0.69	–	–
Family history SAH	0.09 (− 0.18 to 0.35)	0.51	–	–
Rupture	0.42 (0.29 to 0.55)	< 0.01	0.35 (0.22 to 0.48)	< 0.01
*Height/width ratio*				
Age at diagnosis	− 0.01 (− 0.01 to − 0.002)	0.001	− 0.004 (− 0.008 to − 6.44 × 10^−6^)	0.05
Alcohol use	− 0.02 (− 0.14 to 0.10)	0.79	–	–
Tobacco use	0.16 (0.04 to 0.27)	< 0.01	0.11 (− 0.003 to 0.23)	0.06
Female	− 0.03 (− 0.18 to 0.12)	0.68	–	–
Hypertension	− 0.08 (− 0.19 to 0.03)	0.17	–	–
Family history aneurysm	− 0.03 (− 0.20 to 0.13)	0.71	–	–
Family history SAH	0.03 (− 0.19 to 0.24)	0.81	–	–
Rupture	0.24 (0.13 to 0.35)	< 0.01	0.22 (0.10 to 0.33)	< 0.01
*Perpendicular height*				
Age at diagnosis	0.002 (− 0.02 to 0.02)	0.83	–	–
Alcohol use	− 0.32 (− 0.86 to 0.22)	0.25	–	–
Tobacco use	0.32 (− 0.22 to 0.85)	0.25	–	–
Female	0.41 (− 0.31 to 1.13)	0.27	–	–
Hypertension	− 0.37 (− 0.91 to 0.16)	0.17	–	–
Family history aneurysm	− 0.82 (− 1.60 to − 0.03)	0.04	− 0.66 (− 1.44 to 0.11)	0.09
Family history SAH	0.73 (− 1.74 to 0.28)	0.16	–	–
Rupture	1.06 (0.54 to 0.58)	< 0.01	0.96 (0.42 to 1.49)	< 0.01
*Flow angle*				
Age at diagnosis	− 0.19 (− 0.32 to − 0.05)	< 0.01	− 0.12 (0.25 to 0.01)	0.08
Alcohol use	1.05 (− 3.11 to 5.21)	0.62	–	–
Tobacco use	0.79 (− 3.26 to 4.83)	0.70	–	–
Female	− 2.75 (− 8.09 to 2.59)	0.31	–	–
Hypertension	− 0.34 (− 4.26 to 3.58)	0.86	–	–
Family history aneurysm	1.91 (− 3.83 to 7.65)	0.51	–	–
Family history SAH	5.96 (− 1.43 to 13.34)	0.11	–	–
Rupture	11.05 (7.23 to 14.88)	< 0.01	10.38 (6.49 to 14.26)	< 0.01

## Discussion

In this study, we demonstrated that irregular, multilobed PCoA aneurysms with larger height/width ratios and larger flow angles were associated with rupture, whereas perpendicular height was inversely associated with rupture in a multivariable model. Of the above parameters, irregularity, the presence of daughter domes, perpendicular height and height/width ratio are dependent on the aneurysm itself while flow angle gives the relationship between the aneurysm and surrounding vasculature. Conversely, age was inversely associated with aspect ratio with a trend towards an inverse association with height/width ratio and flow angle. There was also a trend towards an association of smoking with height/width ratio and a trend towards an inverse association of family history with perpendicular height.

Previous studies have shown an association between aneurysm rupture and irregular and multilobed aneurysms^11,19–28^. It is believed that multilobed aneurysms are to be in a more advanced stage of development with a greater risk of rupture^29^. We found that there is 3.5-fold increase in the association of multilobed aneurysms with rupture compared to non-multilobed aneurysms (80.3% vs. 22.8%) and a threefold increase in the association of irregular PCoA aneurysms with rupture compared to non-irregular aneurysms (69.0% vs. 21.8%). This finding is similar to a recent large consecutive series of 413 PCoA aneurysms with a threefold increase in the association of rupture in irregular aneurysms compared to non-irregular aneurysms^30^. The association with irregularity and rupture has also been found in a large population-based registry study^31^.

We also found flow angle, which represents the angle at which the aneurysm is tilted with respect to the vector of flow through the parent vessel, to be significantly associated with ruptured PCoA aneurysms. Previous studies have found a similar association with larger flow angle associated with rupture status of PCoA aneurysms^29^. It has been hypothesized that an increasing flow angle causes a higher inflow jet into the aneurysm, resulting in growth in the specific direction^32^. The findings of the association of rupture with larger height/width ratio is also consistent with previous studies^33,34^.

Interestingly, higher age is associated with a lower aspect ratio, with a trend towards lower height/width ratio and smaller flow angle, features that were associated with a lower rupture risk. One can postulate that aneurysms with higher risk features were more prone to be discovered earlier in life. Conversely, aneurysms with more benign features may not be detected until later in life when incidentally found which may result in the association of older age with lower risk. Similarly, there is a trend for family history to be associated with lower perpendicular height, perhaps due to increased screening and earlier discovery of aneurysms.

Further analysis of risk factors demonstrated a trend for smoking to be associated with higher height/width ratio in the multivariable models. Previous studies have found an association of smoking with multiple aneurysms, and larger vessel diameter and size ratio^35^. Smoking is an established risk factor for aneurysmal subarachnoid hemorrhage and it has been postulated that cigarette exposure is associated with downstream inflammation, altering matrix metalloproteinases and vascular smooth muscle cells^36^. The effects on inflammation and vascular smooth muscle cells may explain the increased height/width ratio.

The main limitations of our study are due to its retrospective design. Aneurysm rupture could have affected the morphology of the aneurysm. Therefore, all associations in the parameters examined that were related to intrinsic aneurysm morphology may be a result of aneurysm rupture rather than predictors of rupture risk. In addition, smaller ICA diameter was associated with an increased rupture risk, but it is possible that this is the result of a vasoconstrictive response due to rupture. Measurements were performed manually by a neurosurgeon (JZ) and if needed, verified by a second neurosurgeon (RD). The manual rather than automated analysis may have introduced some variability in the results, but it is a much more applicable technique in the clinical setting.

## Conclusions

We showed that irregular, multilobed PCoA aneurysms with larger height/width ratios and larger flow angles were associated with rupture, whereas perpendicular height was inversely associated with rupture. These morphological parameters specific to PCoA aneurysms are practical and straightforward. Assessment of these variables when examining reconstructions of unruptured aneurysms in the clinical setting could contribute to the risk evaluation in these patients. Furthermore, age was inversely associated with aspect ratio with a trend towards an inverse association with height/width ratio and flow angle. There was also a trend towards an association of smoking with larger height/width ratio and a trend towards an inverse association of family history with perpendicular height. The association of clinical factors with aneurysm morphology warrants further investigation.

## Supplementary information

Supplementary information 1.
