# Supplementary material for: Age and morphology of posterior communicating artery aneurysms
Source: Sci Rep. 2020 Jul 14;10:11545. doi: 10.1038/s41598-020-68276-9 (PMC7360743; doi:10.1038/s41598-020-68276-9)
Supplement: Supplementary file 1 — Supplementary information 1. [file 41598_2020_68276_MOESM1_ESM.docx]

**Supplementary Table 1:** Univariate and multivariate regressions for irregularity and presence of daughter dome.

|  | **Univariable** | | **Multivariable** | |
| --- | --- | --- | --- | --- |
|  | **OR (95% CI)** | **P-value** | **OR (95% CI)** | **P-value** |
| **Irregular** | | | | |
| Age at diagnosis | 0.98 (0.97-0.99) | <0.01 | 0.99 (0.98-1.01) | 0.33 |
| Alcohol use | 1.05 (0.71-1.56) | 0.81 | - | - |
| Tobacco use | 1.46 (0.99-2.16) | 0.06 | 1.21 (0.78-1.90) | 0.40 |
| Female | 0.85 (0.51-1.41) | 0.52 | - | - |
| Hypertension | 1.20 (0.82-1.76) | 0.35 | - | - |
| FH aneurysm | 0.88 (0.50-1.53) | 0.64 | - | - |
| FH SAH | 0.62 (0.29-1.29) | 0.20 | - | - |
| Rupture | 8.02 (5.17-12.43) | <0.01 | 7.20 (4.61-11.25) | <0.01 |
| **Daughter dome** | | | | |
| Age at diagnosis | 0.99 (0.97-<1.00) | 0.04 | 1.00 (0.99-1.02) | 0.81 |
| Alcohol use | 1.02 (0.69-1.52) | 0.92 | - | - |
| Tobacco use | 1.30 (0.88-1.92) | 0.19 | - | - |
| Female | 0.77 (0.46-1.30) | 0.33 | - | - |
| Hypertension | 1.31 (0.89-1.92) | 0.17 | - | - |
| FH aneurysm | 0.64 (0.36-1.12) | 0.12 | - | - |
| FH SAH | 0.60 (0.29-1.25) | 0.18 | - | - |
| Rupture | 13.83 (8.70-21.99) | <0.01 | 14.00 (8.71-22.50) | <0.01 |
